# Supplementary material for: Changes in Whole-Blood microRNA Profiles during the Onset and Treatment Process of Cerebral Infarction: A Human Study
Source: Int J Mol Sci. 2020 Apr 28;21(9):3107. doi: 10.3390/ijms21093107 (PMC7246837; doi:10.3390/ijms21093107)
Supplement: Supplementary file 1 [file ijms-21-03107-s001.pdf]

International Journal of
Molecular Sciences

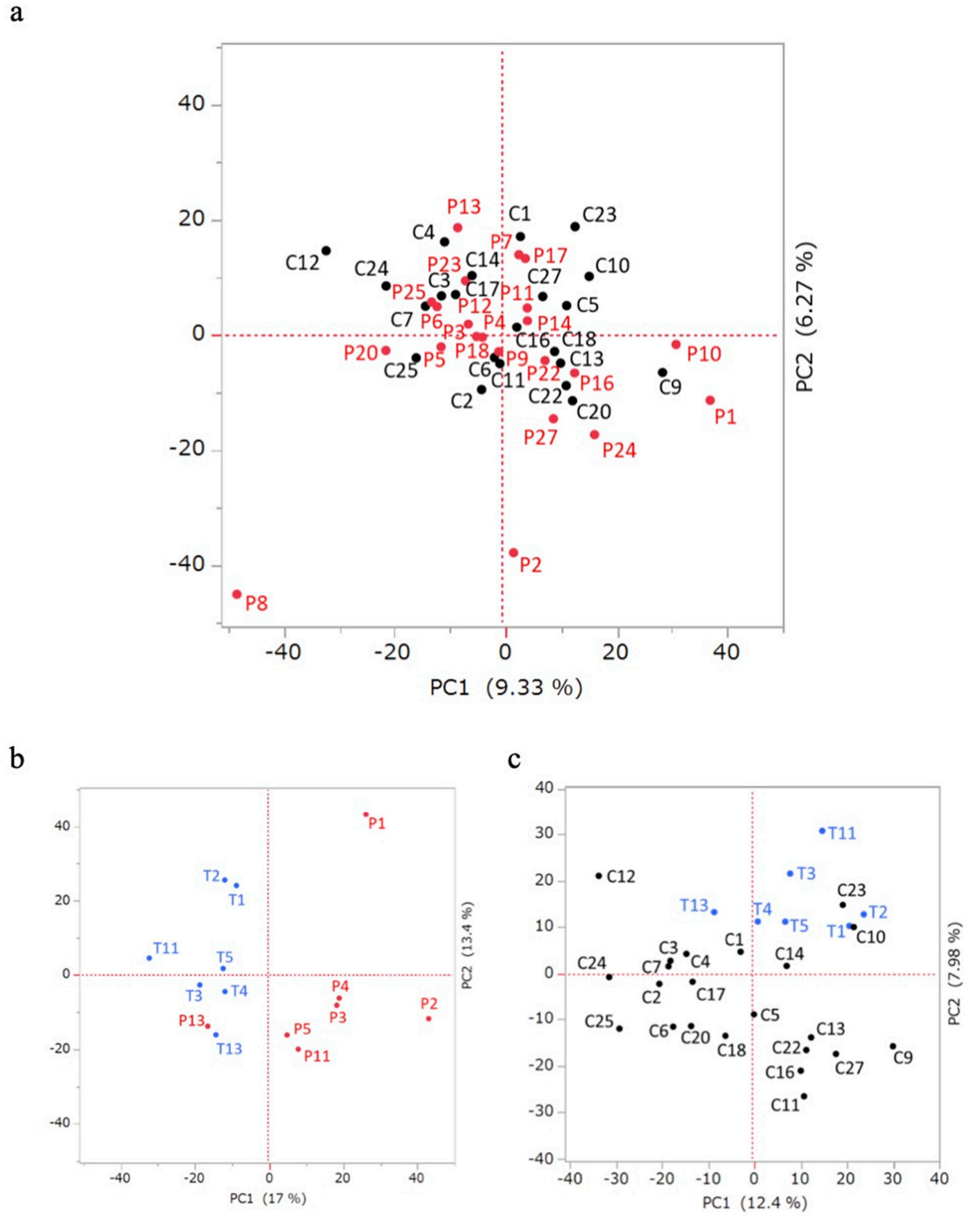

Supplementary Figure S1. Principal component analysis of miRNA expression levels in each group comparison with excluded sample data. The plot for Control and Patient-groups (a), Patient and Treated-groups (b) and Control and Treated-groups (c). "C", "P" or "T" with numbers indicate samples in each group.

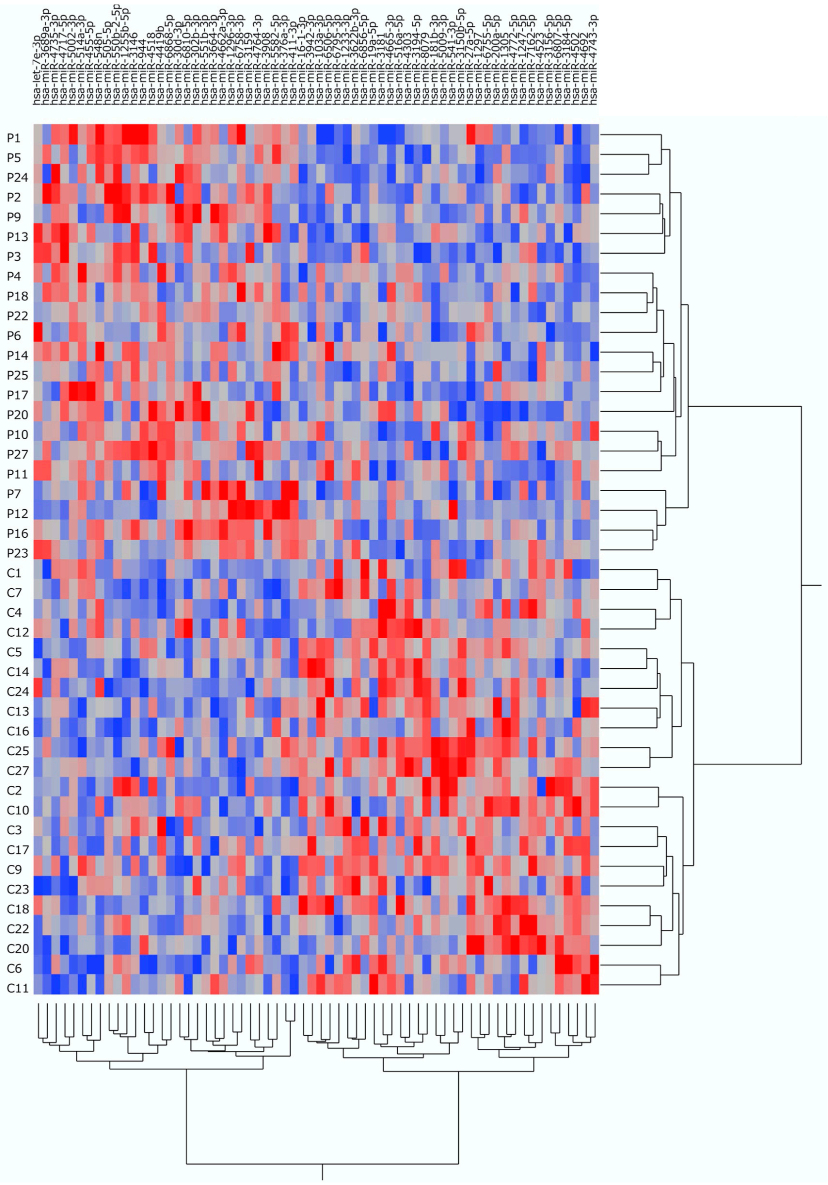

Supplementary Figure S2. Hierarchical clustering analysis of miRNAs expressed differentially between P and C-groups. Expression levels of 30 (C < P) and 34 (C > P) miRNAs (Table 2) were represented in a heatmap with the dendrograms for the subjects and the miRNAs. Names of subjects and miRNAs were indicated at left and top sides, respectively.

© 2020 by the author. Licensee MDPI, Basel, Switzerland. This article is an open access article distributed under the terms and conditions of the Creative Commons Attribution (CC BY) license (http://creativecommons.org/licenses/by/4.0/).
